# Supplementary material for: Systematic review of candidate prognostic factors for falling in older adults identified from motion analysis of challenging walking tasks
Source: Eur Rev Aging Phys Act. 2023 Feb 11;20:2. doi: 10.1186/s11556-023-00312-9 (PMC9921041; doi:10.1186/s11556-023-00312-9)
Supplement: Supplementary file 4 — Additional file 4: Appendix D. Description of the ageing studies, ordered by walking task. [file 11556_2023_312_MOESM4_ESM.docx]

**Appendix D - Description of the ageing studies, ordered by walking task.**

Abbreviations: years old: y; male: m; female: f.

Shading: not reported

Risk of bias: Low, Moderate, Serious


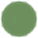


Assessment of participant status:

- Fall history:
  - ✓ if fall history preceding the measurement was assessed
  - 🗶 if fall history was assessed and fallers excluded from the study; in this case, we indicate the older adults as being at low fall risk.
- Physical/mental level:
  - ✓ if physical/mental level was tested
  - 🗶 if physical/mental level was tested and subjects with low (respectively high) fitness were excluded from the study; in this case, we indicate the older adults as being at low (respectively high) risk.

Medicine usage

- ✓ if participants took medication during the measurement period
- 🗶 if participants did not take medication during the measurement period

Measurement devices: if force platforms or infrared cameras were used, but their number was not reported, this is indicated by ✓

Task description: Expected:

- 🗶 if unexpected changes in the task occurred from trial to trail (for example: if various obstacles or stairs were used and these were presented in random order, or if perturbations occurred at random times)
- ✓ otherwise

| **Article** | **Risk of bias** | **Assessment of participant status** | | | **Participants** | | | | | | **Measurement devices** | | **Task description** | | | |
| --- | --- | --- | --- | --- | --- | --- | --- | --- | --- | --- | --- | --- | --- | --- | --- | --- |
|  |  | Fall history (retrospective) | Physical level | Mental level | Community dwelling | Medicine usage | Younger adults | Older adults | Older adults at higher risk | Older adults at lower risk | Force platform | Infrared cameras system | Task | Expected | Self-selected walking velocity | Randomization or fatigue avoidance |
| **Stairs** | | | | | | | | | | | | | | | | |
| Begg (2000) |  |  | 🗶  Questionnaire of musculoskeletal and visual impairments |  | ✓ |  | 6  (21.2 ± 2 y)  (All females) | 6  (67.6 ± 4.8 y)  (All females) |  |  |  | ✓ | Stepping onto raised surface  Height: 15 cm  Length: 500 cm  Width: 100 cm | ✓ | Self-selected | ✓ |
| Bosse (2012) |  |  |  |  | ✓ |  | 13  (25 ± 2 y)  (6 m & 7 f) | 13  (69 ± 4 y)  (7 m 6 f) |  |  | 3 | 13 | Descent  2 steps, each  Height: 17 cm  Width: 30 cm | ✓ | Self-selected |  |
| Chiu (2015) |  |  |  |  |  |  | 20  (25 ± 4.5 y)  (10 m & 10 f) | 20  (74.3 ± 5.9 y, 10 m & 10 f) |  |  |  | 12 | Descent + ascent  4 steps, each  Height: 17 cm  Width: 28 cm | ✓ | Self-selected |  |
| Christina (2002) |  |  | ✓  Visual acuity |  |  | 🗶 | 12  (24 ± 3.3 y)  (5 m & 7 f) | 12  (73.3 ± 1.9 y  (8 m & 4 f) |  |  | 2 |  | Descent + ascent  7 steps, each  Height: 18 cm  Width: 28 cm | ✓ | Fixed velocity  0.65 m/s ± 0.04 m/s | ✓ |
| Crosbie (2003) | 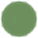 |  | ✓  Melbourne Edge test |  | ✓ |  | 6  (21.9 y)  (3 m & 3 f) | 15  (73.1 y)  (7 m & 8 f) |  |  |  | 1 | Stepping onto and off a raised surface  Height: 9 cm Length: 35 cm Width: 100 cm | ✓ | Self-selected  As fast as possible | ✓ |
| Dewolf (2021) |  | ✓ |  |  |  |  | 8  (28.4 ± 5.2 y)  (4 m & 4 f) |  |  | 10  (73.5 ± 4.5 y)  (9 m & 1 f) |  | 9 | Descent + ascent  2 steps, each  Height: 13 cm  Width: 24.5 cm | ✓ | Self-selected | ✓ |
| Dixon (2018) |  |  | ✓  Timed-Up-and-Go |  |  |  | 20  (24 ± 3 y) | 19  (74.2 ± 6 y) |  |  | 4 | 12 | Descent + ascent  4 steps | ✓ |  |  |
| Francksen (2022) |  |  |  |  |  |  | 27  (24 ± 3 y) | 33  (70 ± 4 y) |  |  | 4 | ✓ | Descent + ascent  7 steps, each  Height: 20 cm  Width: 24 to 26 cm | 🗶 |  | ✓ |
| Francksen (2020) |  |  |  |  | ✓ |  | 26  (24 ± 3y)  No gender information | 33  (70 ± 4y)  No gender information |  |  | 4 | 23 | Descent + ascent  7 steps, each  Height: 20 cm  Width: 24 to 26 cm | 🗶 |  |  |
| Foster (2019) |  |  |  |  | ✓ |  | 17  (25 ± 4 y)  (10 m & 7 f) | 15  (75 ± 3 y)  (5 m & 10 f) |  |  | ✓ | 10 | Descent + ascent  4 steps | ✓ | Self-selected |  |
| Hamel (2005) |  |  | ✓  Visual acuity |  | ✓ | 🗶 | 12  (24.3 ± 2.5 y)  (All females) | 10  (73.5 ± 2.6 y)  (All females) |  |  | ✓ |  | Descent + ascent  7 steps, each  Height: 18 cm  Width: 28 cm | ✓ | Fixed velocity  0.65 m/s ± 0.0325 m/s |  |
| Hsue (2014) |  | 🗶 |  |  | ✓ |  | 28  (<40 y)  (12 m & 16 f) |  |  | 21  (>65 y)  (10 m & 11 f) | 2 | 8 | Descent + ascent  5 steps, each  Height: 18 cm  Width: 28 cm | ✓ | Self-selected | ✓ |
| Hsue (2009) |  |  | ✓  Physical activity questionnaire |  |  |  | 16  (28.7 ± 5.6 y)  (0 m & 16 f) | 10  (70.4 ± 4.4 y)  (0 m & 10 f) |  |  | 2 | 8 | Descent + ascent  5 steps | ✓ | Self-selected | ✓ |
| Kim (2009) |  | ✓ | 🗶  Berg Functional Balance Scale < 50  Frenchay Instrumental Activities of Daily Living score < 50  Physical Function score < 25  Visual acuity | ✓  Mini mental status | ✓ |  | 15  (23.6 ± 2.4 y)  (5 m & 10 f) |  |  | 15  (73.1 ± 4.3 y)  (6 m & 9 f) | ✓ |  | Descent + ascent  3 steps, each  Height: 17 cm  Width: 28 cm | ✓ | Self-selected |  |
| Larsen (2008) |  |  | 🗶  Physical activity questionnaire |  | ✓ |  | 11  (25.8 ± 2 y) |  |  | 19  (72.3 ± 6.6 y) | 1 |  | Descent + ascent  9 steps, each  Height: 16 cm  Width: 23 cm | ✓ | Self-selected |  |
| Mian (2007) |  |  | ✓  Physical activity questionnaire  Short physical performance battery  Timed-Up-and-Go | ✓  Falls efficacy scale | ✓ |  | 13  (28 ± 4 y)  (13 m & 0 f) | 15  (76 ± 3 y)  (15 m & 0 f) |  |  | ✓ | 9 | Descent + ascent  3 steps, each  Height: 16.5 cm  Width: 28 cm | ✓ | 90-95 steps/min |  |
| Novak (2011) |  | 🗶 |  |  | ✓ |  | 24  (23.7 ± 3.0 y)  (7 m & 17 f) |  |  | 33  (67.0 ± 8.2 y)  (14 m & 19 f) | 1 | 2 | Descent + ascent  4 steps, each  Height: 15 cm  Width: 26 cm | ✓ | Self-selected | ✓ |
| Novak (2016) |  |  |  |  | ✓ |  | 14  (25.5 ± 3.2 y)  (8 m & 6 f) | 14  (73.1 ± 6 y)  (7 m & 7 f) |  |  |  | 3 | Descent + ascent  6 steps, each  Height: 7 to 8 inches  Width: 8 to 14 inches | 🗶 | Self-selected | ✓ |
| Reeves (2009) |  |  |  |  |  |  | 17  (24.6 ± 4.1 y)  (10 m & 7 f) | 15  (74.8 ± 2.8 y)  (5 m & 10 f) |  |  | 2 | 9 | Ascent  4 steps, each  Height: 17 cm  Width: 28 cm | ✓ | Self-selected |  |
| **Perturbations** | | | | | | | | | | | | | | | | |
| Afschrift (2019) |  | ✓ |  |  |  |  | 18  (21 ± 2 y) |  |  | 10  (71 ± 4 y) | ✓ | 12 | Anteroposterior and mediolateral support translation | 🗶 | 1.1 m/s | ✓ |
| Bosquée (2021) |  |  |  |  |  |  | 12  (24 ± 3 y) | 11  (72 ± 5 y) |  |  | 1 |  | Backward ankle pull | 🗶 | Self-selected | ✓ |
| Debelle (2021) |  |  |  |  |  |  | 17  (25.2 ± 3.7 y)  (8 m & 9 f) | 17  (62.4 ± 6.6 y)  (3 m & 14 f) |  |  | ✓ | 12 | Backward support acceleration at 5 m/s^2^ | 🗶 | Fixed velocity 1.2  m/s | ✓ |
| Jeon (2022a) |  |  |  | 🗶  Mini mental status < 26 |  |  | 10  (28.2 ± 4.8 y)  (4 m & 6 f) | 10  (62.5 ± 4.7 y)  (7 m & 3 f) |  |  |  | 8 | Anteroposterior support acceleration at 7.75 m/s^2^, 12 m/s^2^, 16.75 m/s^2^ | 🗶 | Self-selected | ✓ |
| Jeon (2022b) |  |  |  |  |  |  | 10  (24 ± 3 y)  (4 m & 6 f) |  |  | 10  (77 ± 8 y)  (5 m & 5 f) |  | 10 | 8 cm surface drop | 🗶 | Self-selected |  |
| Kazanski (2020) |  |  | ✓  Timed-Up-and-Go Square Step Test | ✓  Mini mental status examination  10-point abbreviated Iconographic-Falls Efficacy Scale |  |  | 17  (23.7 ± 3.7 y)  (8m & 9 f) | 17  (67.5 ± 4.9 y)  (7m & 10 f) |  |  |  | 10 | Mediolateral support or visual field translation | ✓ | Self-selected | ✓ |
| Laudani (2021) |  |  | 🗶  Physical activity questionnaire (exclude highly active individuals)  ✓  Berg Balance Scale |  | ✓ |  | 10  (25 ± 2 y)  (2 m & 8 f) |  | 10  (73 ± 5 y)  (2 m & 8 f) |  | 2 | 13 | Mediolateral waist-pull at 10% of the body mass | 🗶 | Self-selected | ✓ |
| Liu (2009) |  |  |  |  | ✓ |  | 9  (23.6 ± 4.8 y) | 9  (73.6 ± 4.4 y) |  |  | 2 | 6 | One of the force plates was covered in soapy water | 🗶 |  |  |
| Martelli (2017) |  | 🗶 |  |  |  | 🗶 | 8  (24 ± 2.7 y) (4 m & 4 f) |  |  | 8  (65 ± 4.8 y)  (5 m & 3 f) |  | 6 | Forward support acceleration at 0.89 m/s^2^, 1.26 m/s^2^, 1.54 m/ s^2^ | 🗶 | Fixed  velocity  V = $\sqrt{Fr}*g*L$,  Fr = 0.15 | ✓ |
| McCrum (2016) |  |  |  |  |  |  | 11  (25.5 ± 2.1 y) (0 m & 11 f) | 14  (69.0 ± 4.7 y)  (0 m & 14 f) |  |  |  | 8 | Backward ankle pull of 2.1 kg | 🗶 | 1.4 m/s |  |
| Mclntosh (2016) |  |  | ✓  Berg Balance Scale |  | ✓ |  | 11  (23.8 ± 3.1 y)  (4 m & 7 f) | 10  (71.1 ± 3.1 y)  (2 m & 8 f) |  |  |  | ✓ | Forward support translation of 16 cm at  130 cm/s^2^ acceleration | 🗶 | Self-selected | ✓ |
| Nachmani (2020) |  |  |  | ✓  Mini mental status | ✓ |  | 19  (26 ± 0.8 y)  No gender information | 35  (81 ± 4.5 y)  No gender information |  |  |  | 2 | Medial-lateral support translation | 🗶 | Self-selected | ✓ |
| Qiao (2018) |  | 🗶 | 🗶  Health questionnaire  BMI > 30 |  |  |  | 11  (24.8 ± 3.4 y)  (5 m & 6 f) |  |  | 11  (75.3 ± 5.4 y)  (5 m & 6 f) |  | 14 | Mediolateral visual field translation | 🗶 | Self-selected | ✓ |
| Ren (2022) |  | 🗶 | ✓  Timed-Up-and-Go |  |  |  | 15  (26.5 ± 3 y) |  |  | 15  (68.3 ± 3.3 y) | 2 | 10 | Anteroposterior support acceleration at 3 m/s^2^ | 🗶 | Self-selected | ✓ |
| Roeles (2018) |  |  | ✓  20 minute walking test |  | ✓ |  | 9  (25.1 ± 3.4 y)  (6 m & 3 f) | 9  (70.1 ± 8.1 y)  (2 m & 7 f) |  |  |  | 32 | Mediolateral or anteroposterior support translations, or room darkening | 🗶 | Self-selected | ✓ |
| Rum (2020) |  |  | ✓  Berg Balance Scale | ✓  Falls Efficacy Scale-International | ✓ | 🗶 | 10  (25 ± 2 y)  (2 m & 8 f) | 10  (73 ± 5y)  (2 m & 8 f) |  |  | 2 | 13 | Mediolateral waist-pull at 10% of the body mass | 🗶 | Self-selected | ✓ |
| Shulman (2018) |  |  | ✓  Waterloo Footedness questionnaire |  | ✓ |  | 18  (21.7 ± 2.6 y)  (10 m & 8 f) | 16  (75.6 ± 5.3 y)  (9m & 7f) |  |  | 2 | 12 | Medial-lateral support translation at 60 cm/s, 2 m/s^2^ | 🗶 |  | ✓ |
| Shulman (2019) |  |  |  |  | ✓ |  | 18  (21.7 ± 2.6 y)  (10 m & 8 f) | 16  (75.6 ± 5.3 y)  (9 m & 7 f) |  |  | 2 | 12 | Anterior-posterior support translation at 60 cm/s, 2 m/s^2^ | 🗶 |  | ✓ |
| Tropea (2015) |  |  |  |  |  |  | 6  (24 ± 1.7 y)  (4 m & 2 f) | 6  (66.7 ± 5.4 y)  (2 m & 4 f) |  |  | ✓ | ✓ | Forward support translation at 8 m/s^2^ | 🗶 | Fixed  velocity  V = $\sqrt{Fr}*g*L$,  Fr = 0.15 |  |
| Yoo (2021) |  |  |  | ✓  Montreal Cognitive Assessment |  |  | 14  (23.4 ± 2.9 y)  (7 m & 7 f) | 14  (70.9 ± 4.5 y)  (4 m & 10 f) |  |  | 2 | 12 | 10 m/s^2^ forward support translation (split-belt treadmill) | 🗶 | Self-selected | ✓ |
| **Obstacles** | | | | | | | | | | | | | | | | |
| Caetano (2016) |  |  | ✓  Melbourne Edge Test  Physiological Performance Assessment  Trail Making Test | ✓  Montreal Cognitive Assessment  Iconographical-Fall Efficacy Scale | ✓ |  | 21  (26 ± 4 y)  (9 m & 12 f) |  |  | 50  (74 ± 7 y)  (16 m & 34 f) | ✓ |  | Visually projected obstacle  Height: 0  Length: between 1/3 of step length and 21.5 cm  Width: < 21.5 cm | ✓ | Self-selected | ✓ |
| Chen (1991) | 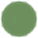 | ✓ | ✓  Visual acuity, osteoarthritic symptoms, hearing loss, lower extremity weakness and reflexes, vibration sense | ✓  Mini mental status | ✓ |  | 24  (21.7 ± 2.1 y)  (12 m & 12 f) |  |  | 24  (71.2 ± 5.5 y)  (12 m & 12 f) |  | 4 | Height: 2.5, 5.1, 15.2 cm | ✓ | Self-selected | ✓ |
| Chen (1994) |  |  | ✓  Visual acuity, pain or numbness, hearing loss, vibratory sense, lower extremity reflexes |  | ✓ | 🗶 | 24  (23.7 ± 2.1 y)  (12 m & 12 f) | 24  (73 ± 5.5 y)  (12 m & 12 f) |  |  |  | ✓ | Visually projected obstacle  Height: 0  Length: 3 cm  Width: 70 cm | ✓ | Self-selected | ✓ |
| Chien (2018) |  | 🗶 |  |  | ✓ |  | 10  (28.1 ± 1 y)  (4 m & 6 f) |  |  | 10  (66.7 ± 5.21 y)  (3 m & 7 f) |  | 8 | Height: 10 % of leg length  Length: 2 cm  Width: 60 cm | ✓ | Self-selected | ✓ |
| Draganich (2004) |  |  |  |  |  |  | 10  (25.9 y)  (5 m & 5 f) | 10  (71.6 y)  (7 m & 3 f) |  |  | 1 | ✓ | Height: 20 cm  Length: 0.5 cm  Width: 90 cm | ✓ | Self-selected  <0.85 m/s  <1.2 m/s | ✓ |
| Eyal (2020) |  | 🗶 | ✓  Four step square test  Mini balance evaluation system  Timed-Up-and-Go | ✓  Montreal cognitive assessment  Color trails test |  |  | 20  (20-35 y)  (10 m & 10 f) |  |  | 20  (70-85 y)  (10 m & 10 f) |  |  | Height: 0.20, 0.5, 0.75, 1, 1.25 cm  Length: 20 cm Width: 60 cm | 🗶 | Self-selected | ✓ |
| Hahn (2004) |  |  | ✓  Berg Balance Scale | ✓  Mini mental status | ✓ |  | 13  (25.7 ± 3.6 y)  (7 m & 6 f) | 13  (72.8 ± 6 y)  (8 m & 5 f) |  |  |  | 6 | Height: 2.5 %, 5%, 10%, 15% of body height | ✓ | Self-selected | ✓ |
| Huang (2008) |  |  |  |  |  |  | 10  (26.1 ± 2.5 y) | 15  (72 ± 6 y) |  |  | 2 | 7 | Height: 10 %, 20%, 30% of leg length | ✓ | Self-selected |  |
| Kim (2013) |  | ✓ | ✓  Berg Balance Scale  Frenchay Activities Index  Physical Functioning score  Health Surveys | ✓  Mini mental status | ✓ | 🗶 | 9  (27 ± 3.6 y)  (4 m & 5 f) |  |  | 9  (75.1 ± 6.7y  (4 m & 5 f) | 2 |  | Height: 10 cm | ✓ | Self-selected | ✓ |
| Kulkarni (2021) |  |  |  |  |  |  | 17  (20.9 ± 1.9 y)  (3 m & 14 f) | 14  (69.7 ± 5.4y) (4 m & 10 f) |  |  |  | ✓ | Height: 23.5 cm  Length: 0.8 cm Width: 100 cm | ✓ | Self-selected |  |
| LoJacono (2018) |  |  | ✓  Physical activity questionnaire |  |  |  | 20  (22.5 ± 3.7 y) | 20  (55.6 ± 6 y) |  |  |  | 12 | Height: 0.5 cm  Length: 10 cm  Width: 100 cm | ✓ | Self-selected | ✓ |
| Lowrey (2007) |  |  |  | ✓  Mini mental status | ✓ | 🗶 | 8  (23.1 ± 2 y)  (4 m & 4 f) | 8  (76.1 ± 4.3 y)  (4 m & 4 f) |  |  |  | ✓ | Height: 45% of lower leg length  Length: 2.5 or 5 cm (unclear) | ✓ | Self-selected | ✓ |
| Lu (2006) |  |  |  |  |  |  | 15  (23 ± 3 y)  No gender information | 15  (72 ± 6 y)  No gender information |  |  | 2 | 7 | Height: 10%, 20% and 30% of leg length | ✓ | Self-selected |  |
| Luo (2022) |  | 🗶 |  |  | ✓ |  | 11  (27 ± 3 y)  (All males) |  |  | 7  (67.5 ± 2.5 y)  (All males) |  | ✓ | Height: 14 cm | ✓ | Self-selected | ✓ |
| Maidan (2018) |  |  | ✓  Montreal cognitive assessment  Trail making test  Four square  step test  Timed-Up-and-Go | 🗶  Mini mental status < 24 | ✓ |  | 20  (29.3 ± 8.8 y)  (10 m & 10 f) | 20  (77.7 ± 3.5 y)  (10 m & 10 f) |  |  |  | ✓ | Height: 2.5, 7.5 cm  Length: 20 cm  Width: 60 cm | 🗶 | Self-selected | ✓ |
| McFadyen (2002) |  |  | ✓  Foot vibration  Ostwestry back-pain questionnaire |  | ✓ |  | 10  (28.4 ± 5.4 y)  (All males) | 10  (69.5 ± 6.1 y)  (All males) |  |  | 2 | ✓ | Height: 5 cm  Length: 11.75 cm  Width: 122 cm | ✓ | Self-selected | ✓ |
| Mckenzie (2004) |  | ✓ | ✓  Standard sensorimotor tests | ✓  Mini mental status |  |  | 15  (22.5 ± 2.77 y)  (5 m & 10 f) |  |  | 17  (68.94 ± 4.85 y)  (7 m & 10 f) |  | 6 | Height: 15 cm  Width: 60, 15 cm | ✓ | Self-selected | ✓ |
| Park (2012) |  |  | ✓  Berg Balance Scale | ✓  Mini mental status |  |  | 9  (25.8 ± 2.8 y)  (3 m & 6 f) | 9  (69.7 ± 3.9 y)  (3 m & 6 f) |  |  |  | 6 | Height: 10%, 20%, 30% of leg length  Width: 100 cm | ✓ | Self-selected | ✓ |
| Tomar (2012) |  |  | ✓  Berg Balance Scale  Trail-making test  Timed-Up-and-Go Choice stepping reaction time | ✓  Mini mental status |  |  | 30  (18-30 y)  (All males) | 30  (>65 y)  (All males) |  |  |  | 1 | Height: 7.6, 12.7, 20.3 cm  Length: 12.7 cm  Width: 25.4 cm | ✓ | Self-selected |  |
| Uchiyama (2012) |  |  | ✓  Berg Balance Scale |  |  |  | 17  (21 ± 2.4 y) | 30  (70 ± 6.9 y) |  |  |  |  | Height: 5 cm Length: 10 cm  Width: 120 cm | ✓ | Self-selected | ✓ |
| Wang (2010) |  |  | ✓  Snellen vision test |  |  | 🗶 | 15  (23 ± 3 y)  (8 m & 7 f) | 15  (72 ± 6 y)  (8 m & 7 f) |  |  | 2 | 7 | Height: 10%, 20% and 30% of leg length | ✓ | Self-selected |  |
| Weerdesteyn (2005a) |  | ✓ |  |  |  |  | 25  (20-37 y)  (4 m & 21 f) |  | 99  (65-88 y)  (22 m & 77 f)  Only subjects with fall history were included |  |  | ✓ | Height: 1.5 cm  Length: 30 cm  Width: 40 cm | 🗶 | Fixed velocity  0.83 m/s | ✓ |
| Weerdesteyn (2005b) |  |  | Recruited from elderly sports groups |  |  |  | 10  (19-32 y)  (0 m & 10 f) |  |  | 10  (65-78 y)  (0 m & 10 f) |  | 6 | Height: 1.5 cm  Length: 30 cm  Width: 40 cm | 🗶 | Self-selected | ✓ |
