# Supplementary material for: Systematic review of candidate prognostic factors for falling in older adults identified from motion analysis of challenging walking tasks
Source: Eur Rev Aging Phys Act. 2023 Feb 11;20:2. doi: 10.1186/s11556-023-00312-9 (PMC9921041; doi:10.1186/s11556-023-00312-9)
Supplement: Supplementary file 5 — Additional file 5: Appendix E. References of ageing studies. [file 11556_2023_312_MOESM5_ESM.docx]

**Appendix E – References of ageing studies**

Afschrift, M., van Deursen, R., De Groote, F., & Jonkers, I. (2019). Increased use of stepping strategy in response to medio-lateral perturbations in the elderly relates to altered reactive tibialis anterior activity. Gait & Posture, 68, 575‑582. https://doi.org/10.1016/j.gaitpost.2019.01.010

Begg, R. K., & Sparrow, W. A. (2000). Gait characteristics of young and older individuals negotiating a raised surface : Implications for the prevention of falls. Journals of Gerontology - Series A Biological Sciences and Medical Sciences, 55(3), 147‑154. https://doi.org/10.1093/gerona/55.3.M147

Bosquée, J., Werth, J., Epro, G., Hülsdünker, T., Potthast, W., Meijer, K., Ellegast, R., & Karamanidis, K. (2021). The ability to increase the base of support and recover stability is limited in its generalisation for different balance perturbation tasks. European Review of Aging and Physical Activity, 18(1), 1‑10. https://doi.org/10.1186/s11556-021-00274-w

Bosse, I., Oberländer, K. D., Savelberg, H. H., Meijer, K., Brüggemann, G. P., & Karamanidis, K. (2012). Dynamic stability control in younger and older adults during stair descent. Human Movement Science, 31(6), 1560‑1570. https://doi.org/10.1016/j.humov.2012.05.003

Caetano, M. J. D., Lord, S. R., Schoene, D., Pelicioni, P. H. S., Sturnieks, D. L., & Menant, J. C. (2016). Age-related changes in gait adaptability in response to unpredictable obstacles and stepping targets. Gait and Posture, 46, 35‑41. https://doi.org/10.1016/j.gaitpost.2016.02.003

Chen, H. C., Ashton-Miller, J. A., Alexander, N. B., & Schultz, A. B. (1991). Stepping over obstacles : Gait patterns of healthy young and old adults. Journals of Gerontology, 46(6), 196‑203. https://doi.org/10.1093/geronj/46.6.M196

Chen, H. C., Ashton-Miller, J. A., Alexander, N. B., & Schultz, A. B. (1994). Effects of age and available response time on ability to step over an obstacle. Journal of Gerontology, 49(5), M227-233. https://doi.org/10.1093/geronj/49.5.m227

Chien, J. H., Post, J., & Siu, K. C. (2018). Effects of Aging on the Obstacle Negotiation Strategy while Stepping over Multiple Obstacles. Scientific Reports, 8(1), 1‑9. https://doi.org/10.1038/s41598-018-26807-5

Chiu, S. L., Chang, C. C., Dennerlein, J. T., & Xu, X. (2015). Age-related differences in inter-joint coordination during stair walking transitions. Gait and Posture, 42(2), 152‑157. https://doi.org/10.1016/j.gaitpost.2015.05.003

Christina, K. A., & Cavanagh, P. R. (2002). Ground reaction forces and frictional demands during stair descent : Effects of age and illumination. Gait and Posture, 15(2), 153‑158. https://doi.org/10.1016/S0966-6362(01)00164-3

Crosbie, J., & Gan, N. (2003). Effect of age and visual contrast on gait during obstacle negotiation. Australasian Journal on Ageing, 22(3), 131‑135. https://doi.org/10.1111/j.1741-6612.2003.tb00483.x

Debelle, H., Maganaris, C. N., & O’Brien, T. D. (2021). Biomechanical Mechanisms of Improved Balance Recovery to Repeated Backward Slips Simulated by Treadmill Belt Accelerations in Young and Older Adults. Frontiers in Sports and Active Living, 3(September), 1‑15. https://doi.org/10.3389/fspor.2021.708929

Dewolf, A. H., Sylos-Labini, F., Cappellini, G., Zhvansky, D., Willems, P. A., Ivanenko, Y., & Lacquaniti, F. (2021). Neuromuscular Age-Related Adjustment of Gait When Moving Upwards and Downwards. Frontiers in Human Neuroscience, 15(October), 1‑14. https://doi.org/10.3389/fnhum.2021.749366

Dixon, P. C., Stirling, L., Xu, X., Chang, C. C., Dennerlein, J. T., & Schiffman, J. M. (2018). Aging may negatively impact movement smoothness during stair negotiation. Human Movement Science, 60, 78‑86. https://doi.org/10.1016/j.humov.2018.05.008

Draganich, L. F., & Kuo, C. E. (2004). The effects of walking speed on obstacle crossing in healthy young and healthy older adults. Journal of Biomechanics, 37(6), 889‑896. https://doi.org/10.1016/j.jbiomech.2003.11.002

Eyal, S., Kurz, I., Mirelman, A., Maidan, I., Giladi, N., & Hausdorff, J. M. (2020). Successful Negotiation of Anticipated and Unanticipated Obstacles in Young and Older Adults : Not All Is as Expected. Gerontology, 66(2), 187‑196. https://doi.org/10.1159/000502140

Foster, R. J., Maganaris, C. N., Reeves, N. D., & Buckley, J. G. (2019). Centre of mass control is reduced in older people when descending stairs at an increased riser height. Gait & Posture, 73, 305‑314. https://doi.org/10.1016/j.gaitpost.2019.08.004

Francksen, N., Ackermans, T., Holzer, D., Maganaris, C., Hollands, M., Roys, M., & O’Brien, T. (2022). Underlying mechanisms of fall risk on stairs with inconsistent going size. Applied ergonomics, 101, e103678‑e103678. https://doi.org/10.1016/j.apergo.2022.103678

Francksen, N. C., Ackermans, T. M. A., Holzer, D., Ebner, S. A., Maganaris, C. N., Hollands, M. A., Karamanidis, K., Roys, M., & O’Brien, T. D. (2020). Negotiating stairs with an inconsistent riser : Implications for stepping safety. Applied Ergonomics, 87(July 2019), 103131. https://doi.org/10.1016/j.apergo.2020.103131

Hahn, M. E., & Chou, L.-S. (2004). Age-related reduction in sagittal plane center of mass motion during obstacle crossing. Journal of Biomechanics, 37(6), 837‑844. https://doi.org/10.1016/j.jbiomech.2003.11.010

Hamel, K. A., Okita, N., Bus, S. A., & Cavanagh, P. R. (2005). A comparisson of foot/ground interaction during stair negotiation and level walking in young and older women. Ergonomics, 48(8), 1047‑1056. https://doi.org/10.1080/00140130500193665

Hsue, B. J., & Su, F. C. (2009). Kinematics and kinetics of the lower extremities of young and elder women during stairs ascent while wearing low and high-heeled shoes. Journal of Electromyography and Kinesiology, 19(6), 1071‑1078. https://doi.org/10.1016/j.jelekin.2008.09.005

Hsue, B.-J., & Su, F.-C. (2014). Effects of Age and Gender on Dynamic Stability During Stair Descent. Archives of Physical Medicine and Rehabilitation, 95(10), 1860‑1869. https://doi.org/10.1016/j.apmr.2014.05.001

Huang, S. C., Lu, T. W., Chen, H. L., Wang, T. M., & Chou, L. S. (2008). Age and height effects on the center of mass and center of pressure inclination angles during obstacle-crossing. Medical Engineering and Physics, 30(8), 968‑975. https://doi.org/10.1016/j.medengphy.2007.12.005

Jeon, W., Wang, S., Bhatt, T., & Westlake, K. P. (2022a). Perturbation-Induced Protective Arm Responses : Effect of Age, Perturbation-Intensity, and Relationship with Stepping Stability : A Pilot Study. Brain Sciences, 12(7). https://doi.org/10.3390/brainsci12070953

Jeon, W., Whitall, J., & Westlake, K. (2022b). Age-related differences in stepping stability following a sudden gait perturbation are associated with lower limb eccentric control of the perturbed limb. Experimental Gerontology, 167(August), 111917. https://doi.org/10.1016/j.exger.2022.111917

Kazanski, M. E., Cusumano, J. P., & Dingwell, J. B. (2020). How healthy older adults regulate lateral foot placement while walking in laterally destabilizing environments. Journal of Biomechanics, 104, 109714. https://doi.org/10.1016/j.jbiomech.2020.109714

Kim, H. D. (2009). A comparison of the center of pressure during stair descent in young and healthy elderly adults. Journal of Physical Therapy Science, 21(2), 129‑134. https://doi.org/10.1589/jpts.21.129

Kim, K.-M., Hart, J. M., & Hertel, J. (2013). Influence of body position on fibularis longus and soleus Hoffmann reflexes. Gait & Posture, 37(1), 138‑140. https://doi.org/10.1016/j.gaitpost.2012.06.009

Kulkarni, A., Cho, H. Y., Rietdyk, S., & Ambike, S. (2021). Step length synergy is weaker in older adults during obstacle crossing. Journal of Biomechanics, 118, 110311. https://doi.org/10.1016/j.jbiomech.2021.110311

Larsen, A. H., Puggaard, L., Hämäläinen, U., & Aagaard, P. (2008). Comparison of ground reaction forces and antagonist muscle coactivation during stair walking with ageing. Journal of Electromyography and Kinesiology, 18(4), 568‑580. https://doi.org/10.1016/j.jelekin.2006.12.008

Laudani, L., Rum, L., Valle, M. S., Macaluso, A., Vannozzi, G., & Casabona, A. (2021). Age differences in anticipatory and executory mechanisms of gait initiation following unexpected balance perturbations. European Journal of Applied Physiology, 121(2), 465‑478. https://doi.org/10.1007/s00421-020-04531-1

Liu, J., & Lockhart, T. E. (2009). Age-related joint moment characteristics during normal gait and successful reactive-recovery from unexpected slip perturbations. Gait & Posture, 30(3), 276‑281. https://doi.org/10.1016/j.gaitpost.2009.04.005

Lojacono, B. C. T., Macpherson, R. P., Kuznetsov, N. A., Raisbeck, L. D., Ross, E., Rhea, C. K., & Raisbeck, L. D. (2018). Journal of Motor Learning and Development, 6 (2), 234-249.

Lowrey, C. R., Watson, A., & Vallis, L. A. (2007). Age-related changes in avoidance strategies when negotiating single and multiple obstacles. Experimental Brain Research, 182(3), 289‑299. https://doi.org/10.1007/s00221-007-0986-0

Lu, T. W., Chen, H. L., & Chen, S. C. (2006). Comparisons of the lower limb kinematics between young and older adults when crossing obstacles of different heights. Gait and Posture, 23(4), 471‑479. https://doi.org/10.1016/j.gaitpost.2005.06.005

Luo, Y., Yang, F., Yerebakan, M. O., Zhang, J., & Hu, B. (2022). Load Carriage Modes and Limb Crossing Patterns Altered Gait during Obstacle Negotiation. Journal of Motor Behavior, 54(5), 525‑536. https://doi.org/10.1080/00222895.2021.2017837

Maidan, I., Eyal, S., Kurz, I., Geffen, N., Gazit, E., Ravid, L., Giladi, N., Mirelman, A., & Hausdorff, J. M. (2018). Age-associated changes in obstacle negotiation strategies : Does size and timing matter? Gait & Posture, 59, 242‑247. https://doi.org/10.1016/j.gaitpost.2017.10.023

Martelli, D., Aprigliano, F., Tropea, P., Pasquini, G., Micera, S., & Monaco, V. (2017). Stability against backward balance loss : Age-related modifications following slip-like perturbations of multiple amplitudes. Gait and Posture, 53, 207‑214. https://doi.org/10.1016/j.gaitpost.2017.02.002

McCrum, C., Epro, G., Meijer, K., Zijlstra, W., Brüggemann, G.-P., & Karamanidis, K. (2016). Locomotor stability and adaptation during perturbed walking across the adult female lifespan. Journal of Biomechanics, 49(7), 1244‑1247. https://doi.org/10.1016/j.jbiomech.2016.02.051

McFadyen, B. J., & Prince, F. (2002). Avoidance and accommodation of surface height changes by healthy, community-dwelling, young, and elderly men. Journals of Gerontology - Series A Biological Sciences and Medical Sciences, 57(4), B166‑B174. https://doi.org/10.1093/gerona/57.4.B166

McIntosh, E. I., Zettel, J. L., & Vallis, L. A. (2017). Stepping Responses in Young and Older Adults Following a Perturbation to the Support Surface During Gait. Journal of Motor Behavior, 49(3), 288‑298. https://doi.org/10.1080/00222895.2016.1204262

McKenzie, N. C., & Brown, L. A. (2004). Obstacle negotiation kinematics : Age-dependent effects of postural threat. Gait and Posture, 19(3), 226‑234. https://doi.org/10.1016/S0966-6362(03)00060-2

Mian, O. S., Narici, M. V., Minetti, A. E., & Baltzopoulos, V. (2007). Centre of mass motion during stair negotiation in young and older men. Gait & Posture, 26(3), 463‑469. https://doi.org/10.1016/j.gaitpost.2006.11.202

Nachmani, H., Shani, G., Shapiro, A., & Melzer, I. (2020). Characteristics of first recovery step response following unexpected loss of balance during walking : A dynamic approach. Gerontology, 66(4), 362‑370. https://doi.org/10.1159/000505649

Novak, A. C., & Brouwer, B. (2011). Sagittal and frontal lower limb joint moments during stair ascent and descent in young and older adults. Gait and Posture, 33(1), 54‑60. https://doi.org/10.1016/j.gaitpost.2010.09.024

Novak, A. C., Komisar, V., Maki, B. E., & Fernie, G. R. (2016). Age-related differences in dynamic balance control during stair descent and effect of varying step geometry. Applied Ergonomics, 52, 275‑284. https://doi.org/10.1016/j.apergo.2015.07.027

Park, S. Y., & Lee, Y. S. (2012). Kinematics of the lower limbs during obstacle crossings performed by young adults and the elderly. Journal of Physical Therapy Science, 24(10), 941‑944. https://doi.org/10.1589/jpts.24.941

Qiao, M., Feld, J. A., & Franz, J. R. (2018). Aging effects on leg joint variability during walking with balance perturbations. Gait & Posture, 62, 27‑33. https://doi.org/10.1016/j.gaitpost.2018.02.020

Reeves, N. D., Spanjaard, M., Mohagheghi, A. A., Baltzopoulos, V., & Maganaris, C. N. (2009). Older adults employ alternative strategies to operate within their maximum capabilities when ascending stairs. Journal of Electromyography and Kinesiology, 19(2). https://doi.org/10.1016/j.jelekin.2007.09.009

Ren, X., Lutter, C., Kebbach, M., Bruhn, S., Bader, R., & Tischer, T. (2022). Lower extremity joint compensatory effects during the first recovery step following slipping and stumbling perturbations in young and older subjects. BMC Geriatrics, 22(1), 1‑16. https://doi.org/10.1186/s12877-022-03354-3

Roeles, S., Rowe, P. J., Bruijn, S. M., Childs, C. R., Tarfali, G. D., Steenbrink, F., & Pijnappels, M. (2018). Gait stability in response to platform, belt, and sensory perturbations in young and older adults. Medical and Biological Engineering and Computing, 56(12), 2325‑2335. https://doi.org/10.1007/s11517-018-1855-7

Rum, L., Vannozzi, G., Macaluso, A., & Laudani, L. (2021). Neuromechanical response of the upper body to unexpected perturbations during gait initiation in young and older adults. Aging Clinical and Experimental Research, 33(4), 909‑919. https://doi.org/10.1007/s40520-020-01592-2

Shulman, D., Spencer, A., & Ann Vallis, L. (2019). Older adults exhibit variable responses in stepping behaviour following unexpected forward perturbations during gait initiation. Human Movement Science, 63(May 2018), 120‑128. https://doi.org/10.1016/j.humov.2018.11.008

Shulman, D., Spencer, A., & Vallis, L. A. (2018). Age-related alterations in reactive stepping following unexpected mediolateral perturbations during gait initiation. Gait and Posture, 64(August 2017), 130‑134. https://doi.org/10.1016/j.gaitpost.2018.05.035

Tomar, U. S., & Gupta, N. (2012). An observational study of foot lifts asymmetry during obstacle avoidance. Journal of Neurosciences in Rural Practice, 3(3), 324‑327. https://doi.org/10.4103/0976-3147.102614

Tropea, P., Martelli, D., Aprigliano, F., Micera, S., & Monaco, V. (2015). Effects of aging and perturbation intensities on temporal parameters during slipping-like perturbations. Proceedings of the Annual International Conference of the IEEE Engineering in Medicine and Biology Society, EMBS, 2015-Novem, 5291‑5294. https://doi.org/10.1109/EMBC.2015.7319585

Uchiyama, M., Demura, S., & Sugiura, H. (2012). The mobility performance of the elderly before, during and after crossing over an obstacle. Human Movement, 13(4), 297‑302. https://doi.org/10.2478/v10038-012-0034-1

Wang, T. M., Chen, H. L., Hsu, W. C., Liu, M. W., & Lu, T. W. (2010). Biomechanical role of the locomotor system in controlling body center of mass motion in older adults during obstructed Gait. Journal of Mechanics, 26(2), 195‑203. https://doi.org/10.1017/S1727719100003051

Weerdesteyn, V., Nienhuis, B., & Duysens, J. (2005a). Advancing age progressively affects obstacle avoidance skills in the elderly. Human Movement Science, 24(5‑6), 865‑880. https://doi.org/10.1016/j.humov.2005.10.013

Weerdesteyn, V., Nienhuis, B., Mulder, T., & Duysens, J. (2005b). Older women strongly prefer stride lengthening to shortening in avoiding obstacles. Experimental Brain Research, 161(1), 39‑46. https://doi.org/10.1007/s00221-004-2043-6

Yoo, D., An, J., Seo, K. H., & Lee, B. C. (2021). Aging Affects Lower Limb Joint Moments and Muscle Responses to a Split-Belt Treadmill Perturbation. Frontiers in Sports and Active Living, 3(July), 1‑12. https://doi.org/10.3389/fspor.2021.683039
