# Supplementary material for: Systematic review of candidate prognostic factors for falling in older adults identified from motion analysis of challenging walking tasks
Source: Eur Rev Aging Phys Act. 2023 Feb 11;20:2. doi: 10.1186/s11556-023-00312-9 (PMC9921041; doi:10.1186/s11556-023-00312-9)
Supplement: Supplementary file 6 — Additional file 6: Appendix F. Significant and non-significant findings in ageing studies. [file 11556_2023_312_MOESM6_ESM.docx]

**Appendix F**: Significant and non-significant findings in ageing studies

Overview of the reported outcome parameters and corresponding articles reporting either significant or non-significant findings. Studies with a serious risk of bias are in red. Sign., significant; N.S. non-significant.

| **Success and error** | | **Total** | | **Stairs** | | **Perturbations** | | **Obstacles** | |
| --- | --- | --- | --- | --- | --- | --- | --- | --- | --- |
|  | | Sign. | N.S. | Sign. | N.S. | Sign. | N.S. | Sign. | N.S. |
| Stepping error, accuracy, and success | | 6 | 3 |  |  | Nachmani (2020) | Debelle (2021) | Caetano (2016)  Chen (1994)  Kim (2013)  Weerdesteyn (2005a)  Weerdesteyn (2005b) | Eyal (2020)  LoJacono (2018) |
| **Temporal outcomes** | | **Total** | | **Stairs** | | **Perturbations** | | **Obstacles** | |
|  | | Sign. | N.S. | Sign. | N.S. | Sign. | N.S. | Sign. | N.S. |
| Reaction time | | 0 | 1 |  |  |  |  |  | Weerdesteyn (2005a) |
| Cadence | | 1 | 2 |  |  |  | Martelli (2017) | Caetano (2016) | McFadyen (2002) |
| **Foot and head outcomes** | | **Total** | | **Stairs** | | **Perturbations** | | **Obstacles** | |
|  | | Sign. | N.S. | Sign. | N.S. | Sign. | N.S. | Sign. | N.S. |
| Foot velocity | | 0 | 1 |  |  |  |  |  | Luo (2022) |
| Foot velocity variation | | 0 | 1 |  |  |  |  |  | Luo (2022) |
| Head velocity | | 1 | 0 | Dixon (2018) |  |  |  |  |  |
| Power spectrum head velocity | | 1 | 0 | Dixon (2018) |  |  |  |  |  |
| **Center of Mass (CoM) outcomes** | | **Total** | | **Stairs** | | **Perturbations** | | **Obstacles** | |
|  | | Sign. | N.S. | Sign. | N.S. | Sign. | N.S. | Sign. | N.S. |
| Power spectrum CoM velocity | | 1 | 0 | Dixon (2018) |  |  |  |  |  |
| CoM trajectory and jerk score | | 3 | 2 | Dixon (2018) | Bosse (2012) | Laudani (2021) |  | Wang (2010) | Lowrey (2007) |
| Extrapolated CoM | | 2 | 1 | Bosse (2012) | Novak (2016) | Afschrift (2019) |  |  |  |
| **Force plate outcomes** | | **Total** | | **Stairs** | | **Perturbations** | | **Obstacles** | |
|  | | Sign. | N.S. | Sign. | N.S. | Sign. | N.S. | Sign. | N.S. |
| CoP displacement and velocity | | 3 | 1 | Kim (2009) |  | Afschrift (2019) Jeon (2022b) | Rum (2020) |  |  |
| Base of Support | | 1 | 1 |  |  | Bosquée (2021) | McCrum (2016) |  |  |
| Support moment peak and variability | | 1 | 0 | Novak (2011) |  |  |  |  |  |
| **Dynamic stability outcomes** | | **Total** | | **Stairs** | | **Perturbations** | | **Obstacles** | |
|  | | Sign. | N.S. | Sign. | N.S. | Sign. | N.S. | Sign. | N.S. |
| Angle between CoM and CoP | | 1 | 0 | Huang (2008) |  |  |  |  |  |
| Distance between CoM and BoS | | 1 | 0 |  |  | Nachmani (2020) |  |  |  |
| Relative MoS to baseline | | 1 | 0 |  |  | McCrum (2016) |  |  |  |
| CoM-CoP distance | | 5 | 1 | Hsue (2014)  Huang (2008) Reeves (2009) | Mian (2007) |  |  | Hahn (2004)  Wang (2010) |  |
| CoM-CoP angular velocity | | 0 | 1 |  | Huang (2008) |  |  |  |  |
| **Joint and segment outcomes** | | **Total** | | **Stairs** | | **Perturbations** | | **Obstacles** | |
|  | | Sign. | N.S. | Sign. | N.S. | Sign. | N.S. | Sign. | N.S. |
| Ankle | motion | 5 | 3 | Hsue (2009) | Dewolf (2021)  Reeves (2009) |  |  | Chen (1991)  Lu (2006)  McFadyen (2002)  Park (2012) | Draganich (2004) |
|  | motion variability | 1 | 0 |  |  | Qiao (2018) |  |  |  |
|  | moment rate | 1 | 0 |  |  | Liu (2009) |  |  |  |
|  | power | 1 | 0 |  |  |  |  | McFadyen (2002) |  |
|  | work | 0 | 1 |  | Foster (2019) |  |  |  |  |
|  | angular impulse | 1 | 0 | Bosse (2012) |  |  |  |  |  |
| Knee | motion | 5 | 5 | Bosse (2012)  Hsue (2009) | Reeves (2009) | Jeon (2022b) |  | Chien (2018)  Park (2012) | Chen (1991)  Draganich (2004)  Lu (2006)  McFadyen (2002) |
|  | motion variability | 1 | 0 |  |  | Qiao (2018) |  |  |  |
|  | moment rate | 1 | 0 |  |  | Liu (2009) |  |  |  |
|  | power | 0 | 1 |  |  |  | Jeon (2022b) |  |  |
|  | work | 1 | 1 |  | Foster (2019) | Jeon (2022b) |  |  |  |
|  | angular impulse | 1 | 0 | Bosse (2012) |  |  |  |  |  |
|  | power | 1 | 0 |  |  |  |  | McFadyen (2002) |  |
| Hip | motion variability | 1 | 0 |  |  | Qiao (2018) |  |  |  |
|  | moment rate | 1 | 0 |  |  | Liu (2009) |  |  |  |
|  | flexion velocity | 1 | 0 |  |  |  |  | Draganich (2004) |  |
| Ankle, knee and hip coordination patterns | | 1 | 0 | Chiu (2015) |  |  |  |  |  |
| Shank | Range of Motion | 1 | 0 | Dewolf (2021) |  |  |  |  |  |
|  | elevation angle | 0 | 1 |  | Dewolf (2021) |  |  |  |  |
| Thigh | Range of Motion | 1 | 0 | Dewolf (2021) |  |  |  |  |  |
|  | elevation angle | 0 | 1 |  | Dewolf (2021) |  |  |  |  |
| Thigh-shank phase lag | | 1 | 0 | Dewolf (2021) |  |  |  |  |  |
| Arm | displacement | 0 | 1 |  |  |  | Jeon (2022a) |  |  |
|  | displacement velocity | 0 | 1 |  |  | Jeon (2022a) |  |  |  |
| Body tilt | | 2 | 4 | Novak (2016) | Bosse (2012)  Hsue (2009) | Rum (2020) | Debelle (2021)  Martelli (2017) |  |  |
